# Acromion Morphology of Patients with Rotator Cuff Disease in Standard AP Shoulder Radiograph in Hospital Sultanah Bahiyah and Hospital Kuala Lumpur

**DOI:** 10.5704/MOJ.2211.009

**Published:** 2022-11

**Authors:** YC Leong, CW Yeoh, MI Azman, MS Juhari, HT Siti

**Affiliations:** 1 Department of Orthopaedics, Hospital Kuala Lumpur, Kuala Lumpur, Malaysia; 2 Department of Orthopaedics, Hospital Sultanah Bahiyah, Alor Setar, Malaysia

**Keywords:** acromion morphology, radiograph, rotator cuff tear

## Abstract

**Introduction:**

Rotator cuff pathology is commonly attributed to acromion morphology that is demonstrable in standard AP shoulder radiographs by measuring the critical shoulder angle (CSA), the lateral acromial angle (LAA), and the acromial index (AI). However, these parameters vary among races and countries. Therefore, our study aimed to get the local data on acromion morphology in patients with rotator cuff disease.

**Materials and methods:**

MRI shoulder reports between January 2012 and June 2018 were reviewed. The study group consisted of 47 patients with rotator cuff injury with a partial or complete tear, and a control group of 37 patients with tendinitis or osteoarthritis and intact rotator cuffs. The CSA, LAA, and AI of both groups were measured on the anteroposterior shoulder radiograph. The risk factors for both groups and the acromion morphology were recorded.

**Results:**

The CSA for the rotator cuff tear and the control group was 39.08° and 38.28°, LAA was 72.57 ° and 73.51°, and AI was 0.79 and 0.75. The acromion morphology differed in terms of gender, and only LAA was different among the different ethnic groups. There was a negative correlation between age and CSA, age and AI, LAA and CSA, LAA and AI, but a positive correlation between AI and CSA.

**Conclusion:**

The CSA for rotator cuff tear patients in our population was 39.08°, LAA was 72.57°, and AI was 0.79. The acromion morphology was significantly influenced by age and gender.

## Introduction

Rotator cuff disease is the commonest diagnosis for chronic shoulder pain in an aging population^1^. It includes a spectrum of diseases, ranging from impingement syndrome, tendinitis, tears to arthropathy. Both intrinsic and extrinsic factors have been postulated as causes of the disease. Intrinsic factors are mainly degeneration and hypovascularity, leading to collagen fibre abnormalities. Extrinsic factors are subacromial impingement and shoulder overuse^2^.

The acromion is a continuity of the lateral aspect of the scapula spine, palpable at the most lateral aspect of the shoulder. A laterally hooked and curved acromion will cause irritation and rotator cuff tear^2^. The acromion morphology can be measured through plain radiographs, computed tomography (CT), and magnetic resonance imaging (MRI). The plain radiograph is a simple, cheap, and easily available modality in most primary centres. Several acromium parameters including the CSA, LAA, and AI can be assessed through the standard plain anteroposterior radiograph of the shoulder^3-5^.

A previous study reported that the morphology of the scapula differed among races and countries. Cabezas *et al* noted that the East Asian population had smaller acromion morphology, but more laterally extended acromion than the North American^6^. Previous studies found that if the CSA was more than 38°, LAA smaller than 70°, and AI larger than 0.75, there was a higher risk of rotator cuff tear^3-5^.

This was a retrospective study of the acromion morphology among patients with rotator cuff disease treated in Hospital Sultanah Bahiyah and Hospital Kuala Lumpur. The results would be very useful for both primary and tertiary centres as plain radiograph is a readily available and cost-saving modality in the diagnosis of rotator cuff disease. We also aimed to explore the factors that influenced acromion morphology.

## Materials and Methods

All magnetic resonance imaging (MRI) of the shoulder performed in patients 40 years old and above in Hospital Sultanah Bahiyah and Hospital Kuala Lumpur from January 2012 to June 2018 were traced. A total of 108 MRI reports of our patients, validated by our radiologist, was reviewed. There were 82 patients from Hospital Sultanah Bahiyah and 26 patients from Hospital Kuala Lumpur. We excluded 24 patients with glenohumeral joint osteoarthritis, infection, tumour, history of previous fracture, and incomplete data.

**Fig. 1.**
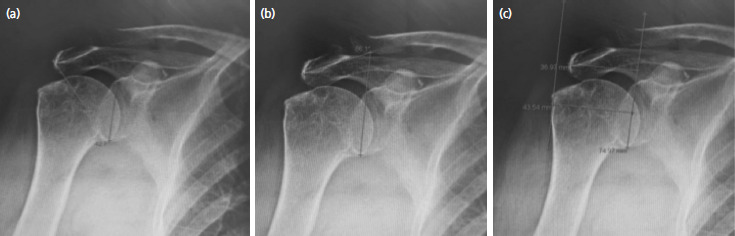
(a) CSA was measured by a line connecting the superior and inferior bony margins of the glenoid and an intersecting line drawn from the inferior bony margin of the glenoid to the most lateral border of the acromion. (b) LAA was measured by drawing a line along the superior- and inferior-most lateral points of the glenoid and an intersecting line drawn parallel to the acromion undersurface. (c) AI was measured by the distance from the glenoid plane to the acromion divided by the distance from the glenoid plane to the lateral aspect of the humeral head.

The study group comprised 47 patients with rotator cuff injuries with partial or complete tear. The control group comprised of 37 patients with normal rotator cuffs. The CSA, LAA, and AI of these patients were measured by using a standard anteroposterior radiograph (Fig. 1). All the patients were kept anonymous, with an individual code for each. All parameters from plain radiographs and MRI reports were recorded by different researchers to prevent bias. Finally, the risk factors for both groups were recorded.

Ethical approval for this study was obtained from the Medical Research and Ethics Committee (MREC), Ministry of Health, Malaysia. We conducted our study in compliance with ethical principles outlined in the Declaration of Helsinki and the Malaysian Good Clinical Practice Guidelines.

Calculations were done using SPSS software version 16.0. Data were expressed as mean, standard deviation as well as frequency and percentage. The means of the critical shoulder angle, lateral acromion angle, and acromion index from each group were compared using the independent t-test or one-way ANOVA test. Age, CSA, LAA and AI were tested for correlation using the Pearson correlation coefficient (PCC) or Spearman correlation coefficient (SCC). The significance level was set at p<0.05.

## Results

The patient demographics in both groups are illustrated in Table I. The mean age for patients with rotator cuff tears was 61.4 years (SD: ±9.47), and for the controls with the intact rotator cuffs was 52.2 years (SD: ±10.02). 51.9% of the male patients, and 63.3% of the female patients had MRI evidence of rotator cuff tear. The incidence of rotator cuff tears for Malay was 58.1%, whereas Chinese was 65%, and Indian was 42.9%. A total of 50% of patients with diabetes mellitus had rotator cuff tears. 62.2% of the non-diabetics did not have a tear. The CSA for rotator cuff tear and the control group was 39.08° (SD: ±4.734°) and 38.28° (SD: ±4.572°), LAA was 72.57° (SD: ±8.037°) and 73.51° (SD: ± 6.423°), and AI was 0.79 (SD: ±0.058) and 0.75 (SD: ±0.077). No significant difference was found between age, gender, ethnicity, diabetes mellitus, and acromion morphology in both groups.

**Table I:** [1] Independent t-test, [2] Pearson chi-square

	zTear (N=47)	Intact (N=37)	p-value
Age (Mean, SD)	61.4 (9.47)	52.2 (10.02)	0.857^1^
Gender			
Male	28 (51.9%)	26 (48.1%)	0.310^2^
Female	19 (63.3%)	11 (36.7%)	
Ethnicity			
Malay	25 (58.1%)	18 (41.9%)	0.331^2^
Chinese	13 (65%)	7 (35%)	
Indian	9 (42.9%)	12 (57.1%)	
Diabetes Mellitus			
Yes	17 (50%)	17 (50%)	0.422^2^
No	28 (62.2%)	17 (37.8%)	
CSA (Mean, SD)	39.08 (4.734)	38.28 (4.572)	0.432^1^
LAA (Mean, SD)	72.57 (8.037)	73.51 (6.423)	0.564^1^
AI (Mean, SD)	0.79 (0.058)	0.75 (0.077)	0.014^1^

Table II showed significant differences in terms of gender with CSA, LAA, and LA, LAA and ethnicity, but no difference between diabetes mellitus and acromion morphology. Table III illustrates the correlation between age, CSA, LAA and AI. There was a negative correlation between the age and CSA; age and AI; AI and LAA; LAA and CSA, but a positive correlation between the AI and CSA.

**Table II:** [1] independent T-test, [2] One way ANOVA

	CSA	p-value	LAA	p-value	AI	p-value
Gender						
Male	37.81 (4.336)	0.014^1^	74.53 (5.936)	0.0091	0.75 (0.063)	0.001^1^
Female	40.38 (4.816)		70.20 (8.798)		0.80 (0.068)	
Ethnicity						
Malay	39.12 (5.038)	0.623^2^	72.33 (7.981)	0.0262	0.77 (0.071)	0.518^2^
Chinese	38.75 (4.028)		70.70 (6.465)		0.78 (0.071)	
Indian	37.90 (4.482)		76.52 (5.564)		0.75 (0.069)	
Diabetes Mellitus						
Yes	39.12(5.038)	0.572^1^	74.38(6.095)	0.1641	0.76(0.059)	0.560^1^
No	38.75(4.028)		72.04(8.127)		0.77(0.074)	

**Table III:** 1CC : Spearman correlation coefficient, 2CC : Pearson correlation coefficient, which was graded as excellent (0.811.00), good (0.61-0.80), moderate (0.41-0.60), fair (0.21=0.40), poor (0.00-0.20)

	CSA	LAA	AI
Age			
1CC	-0.229	0.143	-0.242
p-value	*0.036	0.194	0.027
CSA			
2CC	-	-0.559	0.770
p-value	-	*<0.05	*<0.05
LAA			
2CC	-	-	-0.503
p-value	-	-	*<0.05

## Discussion

Rotator cuff disease is commonly referred to sport and arthroscopy unit. It includes a sequence of impingement syndrome and rotator cuff tear caused by hooked, curved, and laterally extended acromions. These acromion features can be explored by measuring the CSA, LAA and AI in a standard plain anteroposterior radiograph of the shoulder^3-5^.

Moor *et al* first introduced the CSA by measuring an angle formed between the articular surface of the glenoid and a line formed by the most inferior aspect of the glenoid to the most lateral aspect of the acromion^4^. The CSA indicated the acromial coverage and the glenoid inclination. A larger angle represented a laterally extended acromion that led to the impingement syndrome and rotator cuff tear. A study on the Swedish population by Moor *et al* elicited CSA of 35° and more were prone to rotator cuff tears^4^. Miswan *et al* from Malaysia reported a CSA of 39.4° (35.0 - 43.8°) in the rotator cuff tears in his patients^7^. Our study also supported his study in which CSA larger than 39° was associated with rotator cuff tear in the Malaysian population.

The LAA was described by Banas *et al* in 19953. It was formed by an angle between the articular surface of the glenoid and the under surface of the acromion. A smaller LAA indicated a smaller subacromial volume that led to impingement on the rotator cuff^8^. Both Banas and Balke *et al* found that the LAA smaller than 70° was linked to rotator cuff tears^9^. In contrast, our population with LAA of 72.49° had rotator cuff tears.

In 2006, Nyffler *et al* described the acromion index (AI) by dividing the line from the glenoid articular surface to the lateral edge of the acromion and the line from the glenoid articular surface to the lateral aspect of the humeral head. He postulated that the higher AI represented a larger extension of the acromion and that those with an AI higher than 0.73 had rotator cuff tears^5^. Meanwhile, the Brazilian population with rotator cuff disease had AI larger than 0.72, and the Japanese had AI larger than 0.6810. However, our findings were similar to the Vietnamese population, where an AI greater than 0.79 was associated with rotator cuff disease^11^. With these differences between different populations, AI might not be a good predictor for rotator cuff tear^12^.

Aging posed a significant risk factor for rotator cuff disease, as tendon degenerated, calcified, and tore, together with diabetes in which the advanced glycation end products (AGEs) increased intermolecular collagen cross-links resulting in stiffer tendon and a higher risk of a tear^13^. The relation between acromion morphology, age, and diabetes had not been investigated before. Our study found a strong correlation between aging and CSA but did not include patients with glenohumeral osteoarthritis. Moor *et al* found that a CSA of less than 28 was associated with osteoarthritis, whereas more than 35 was associated with rotator cuff tears^4^.

There was limited data exploring the relationship between gender and acromion parameters. A study among the Lebanese population found higher LAA in women but no difference in CSA and AI^14^. In our study, women demonstrated higher CSA and AI with lower LAA, which were the indicators for higher risk of rotator cuff tears. In addition, our study observed that female patients had higher prevalence of rotator cuff tears than male patients (63.3% vs. 51.9%), even though not statistically significant. Heart *et al* proposed that hormonal variation in oestrogens and thyroxin affecting the collagen catabolism was the cause of the higher incidence of tendinopathy among women^15^. An epidemiological study also reported that rotator cuff pathology was more common in women than men: 90 vs. 83 cases per 100,000 person-years; p<0.00116.

The AI, LAA, and CSA were known radiographic parameters of risk factors of rotator cuff disease. Moor *et al* evaluated the predictive power of these parameters. He demonstrated an excellent correlation between the AI and CSA, and a moderate correlation between the LAA and CSA. He concluded that the CSA was the most valuable measurement to distinguish between patients with intact rotator cuff tendons and those with torn rotator cuff tendons^17^. Our study demonstrated a similar result, with a moderate negative correlation between the AI and LAA, LAA and CSA, and a positive correlation between the AI and CSA.

There were several limitations to our study. Our study was performed in two tertiary hospitals in Malaysia, and these findings may not represent populations in other regions. Other radiographic parameters, such as acromiohumeral interval and acromioglenoid angle were not included in our study.

## Conclusion

The CSA for rotator cuff tears in patients in our Malaysian population was 39.08°, LAA was 72.57° and AI was 0.79. The acromion morphology was significantly influenced by age and gender.
